# Editorial: Nutritional status and nutritional support in hospitalized patients

**DOI:** 10.3389/fnut.2026.1998511

**Published:** 2026-09-30

**Authors:** Beata Jabłońska, Aurelio Lo Buglio, Natalia Świątoniowska-Lonc

**Affiliations:** 1 Department of Digestive Tract Surgery, Medical University of Silesia, Katowice, Poland; 2 Department of Medical and Surgical Sciences, University of Foggia, Foggia, Italy; 3 Department of Cardiology, Centre for Heart Diseases, 4th Military Hospital, Wroclaw, Poland

**Keywords:** enteral nutrition, hospital malnutrition, immunonutrition, nutritional screening, nutritional status, oral nutritional supplement, parenteral nutrition, patient outcome

## Hospital malnutrition and nutritional support: risk factors, clinical consequences, and nutritional assessment

Hospital malnutrition remains highly prevalent, affecting an estimated 30–50% of admitted patients. Malnutrition is reported in about 84% of cancer patients. Notably, approximately 20% of oncology patients succumb to the consequences of malnutrition rather than the primary malignancy itself (1–5).

Malnutrition is intricately linked to the primary pathology and a multitude of comorbidities, including gastrointestinal, cardiovascular, respiratory, neurological, and musculoskeletal disorders. Furthermore, nutritional depletion is closely associated with impaired humoral and cellular immune responses. Concurrently, systemic inflammation serves as a critical indicator of poor clinical prognosis in these patients (6–10).

Disease-related malnutrition may result from insufficient food intake due to dysphagia, odynophagia, nausea, vomiting, loss of appetite, as well as impaired digestion and absorption related to the underlying disease. Importantly, invasive procedures, oncological treatments such as radiotherapy and chemotherapy, and some medical therapies may further compromise nutritional intake and nutritional status in hospitalized patients. Moreover, hospitalization itself may contribute to inadequate nutritional intake and increase the risk of malnutrition (11–16).

On the other hand, poor nutritional status adversely affects patient prognosis and overall quality of life. Conversely, timely preoperative nutritional interventions, guided by accurate baseline assessments, can significantly decrease the risk of treatment-related and post-operative complications. Nutritional status should be assessed using a comprehensive approach that combines subjective and objective measures, including anthropometric, clinical, and biochemical parameters (3, 4, 16).

The clinical importance of this issue is supported by substantial evidence linking malnutrition with adverse outcomes across hospital departments. Specifically, poor nutritional status is associated with elevated morbidity and mortality rates, extended hospitalizations, and increased healthcare expenditures. In surgical oncology, nutritional depletion correlates with heightened perioperative morbidity and reduced overall survival. For surgical patients, malnutrition directly contributes to impaired wound healing, coagulopathy, vascular fragility, a higher incidence of infectious complications, and an overall increased rate of post-operative adverse events, all of which prolong hospital stays (17–22).

Although targeted nutritional therapy can effectively improve clinical outcomes, malnutrition remains frequently underdiagnosed and inadequately managed. Consequently, timely and accurate nutritional status evaluation is essential to implement appropriate supportive care. Standard nutritional screening integrates dynamic parameters, such as unintentional weight loss and biochemical indices, including the Prognostic Nutritional Index (PNI), and objective anthropometric data, including body mass index (BMI), fat mass, waist-to-hip ratio, and muscle strength. In clinical practice, the most widely adopted validated screening frameworks include the Subjective Global Assessment (SGA), Nutritional Risk Screening 2002 (NRS 2002), and the Malnutrition Universal Screening Tool (MUST) (23–25).

## Nutritional support: indications, modalities, and clinical applications

Nutritional support is an important component of comprehensive medical care in patients who are unable to meet their nutritional requirements through regular food intake. It may be required across a broad spectrum of clinical conditions, including gastrointestinal diseases, cancer, major surgery, critical illness, inflammatory disorders, neurological diseases, severe infections, and conditions associated with prolonged hospitalization. The primary objective of nutritional support is to prevent or treat disease-related malnutrition, preserve lean body mass and physical function, support immune competence and tissue repair, improve tolerance to disease-specific treatment, and reduce nutrition-related complications. Importantly, nutritional support should not be regarded as a uniform intervention; rather, the modality, timing, composition, route, and intensity of nutritional therapy should be individualized according to nutritional status, disease severity, metabolic requirements, gastrointestinal function, and the patient's ability to consume and absorb nutrients (1, 16, 26–37).

The first step in nutritional support is the optimization of oral nutritional intake. Whenever the patient is able to eat safely and the gastrointestinal tract is functional, oral nutrition should remain the preferred route. Nutritional counseling should focus on increasing energy and protein intake, modifying meal frequency and consistency, and addressing potentially reversible factors limiting food consumption. When dietary intake remains insufficient despite counseling and food modification, oral nutritional supplements (ONS) may be introduced. ONS provide a concentrated source of energy, protein, and micronutrients and may be administered as a supplement to regular meals rather than as their replacement. They are particularly useful in patients with inadequate spontaneous intake, weight loss, increased nutritional requirements, or disease-related anorexia who are still able to swallow and absorb nutrients. ONS may therefore represent an important early intervention in patients with cancer, chronic diseases, gastrointestinal disorders, and during the perioperative period. Overall, ONS are a crucial bridge between standard oral diet and artificial nutrition, effectively preventing further clinical deterioration (1, 16, 38–41). When oral intake is inadequate or impossible and the gastrointestinal tract is functional, enteral nutrition (EN) is generally the preferred form of artificial nutritional support. EN delivers nutrients directly into the gastrointestinal tract through feeding tubes or enteral access devices and allows the physiological functions of the intestine to be maintained. Depending on the anticipated duration and clinical circumstances, EN may be administered through nasogastric or nasojejunal tubes or through more durable access, including gastrostomy or jejunostomy. EN is particularly relevant in patients with severe neurological disorders and dysphagia, head and neck cancer, critical illness, prolonged inadequate oral intake, and selected patients with gastrointestinal and pancreatic diseases. In severe acute pancreatitis, for example, early EN is recommended whenever feasible because it supports intestinal barrier integrity and is associated with a lower risk of infectious complications compared with strategies relying primarily on parenteral nutrition (26, 41–46). Thus, the presence of a gastrointestinal disease does not itself constitute an indication for PN; rather, the functional capacity of the gastrointestinal tract should guide the selection of the nutritional route. Parenteral nutrition is used when nutritional requirements cannot be adequately met through the oral or enteral route or when the gastrointestinal tract cannot be safely or effectively used. PN may be indicated in patients with intestinal obstruction, severe intestinal failure, prolonged gastrointestinal intolerance, severe malabsorption, or other conditions in which adequate EN is impossible or contraindicated. It may also be considered as supplemental PN when EN alone does not provide sufficient nutritional intake over an appropriate period. PN can be administered through peripheral or central venous access depending on the expected duration of treatment and the composition and osmolarity of the nutritional formulation. Because PN is associated with catheter-related, infectious, metabolic, and hepatobiliary complications, its use requires careful prescription, monitoring, and adjustment. Consequently, PN should generally be considered when the oral or enteral route is insufficient or unavailable rather than automatically as the first-line approach (26, 47–50).

An additional aspect of nutritional therapy is immunonutrition (IN), which involves the use of nutrients with proposed immunomodulatory and metabolic effects. Formulations used for immunonutrition may contain combinations of arginine, omega-3 fatty acids, glutamine, nucleotides, and other bioactive substrates. The rationale for their use is based on their potential to modulate excessive inflammatory responses, support immune function, maintain intestinal barrier integrity, and facilitate tissue repair. IN has been investigated particularly in patients undergoing major surgery, including gastrointestinal and pancreatic procedures. Nevertheless, immunonutrition should be considered within the context of the overall nutritional strategy, and its potential benefits should be interpreted according to the clinical setting, patient selection, and composition of the nutritional formula (37, 51, 52).

Alongside macronutrient support to meet protein and caloric requirements, nutritional management should include the systematic correction of deficiencies in macro- and microelements, essential trace elements, and water- and fat-soluble vitamins. These micronutrients serve as mandatory cofactors for cellular metabolism, enzymatic reactions, tissue repair, and immune system competence. In patients at risk of refeeding syndrome, particular attention should be paid to thiamine and electrolyte abnormalities, especially phosphate, potassium, and magnesium, with appropriate supplementation and monitoring (30, 53–56).

Therefore, hospitalized patients across clinical departments and with disorders affecting different physiological systems require systematic assessment of nutritional status and timely, individualized nutritional support (16, 30, 56, 57).

Overall, nutritional support should be considered an active therapeutic intervention applicable across multiple clinical conditions rather than a treatment restricted to patients with gastrointestinal diseases. ONS, EN, and PN constitute the principal modalities of nutritional support, with oral nutrition and ONS preferred whenever adequate intake is possible, EN favored when the gastrointestinal tract is functional but oral intake is insufficient, and PN reserved for situations in which enteral or oral nutrition is contraindicated, impossible, or inadequate. Immunonutrition represents a more specialized strategy that may be considered in selected clinical settings, particularly in major surgical patients. The central principle is therefore not simply to provide nutrients, but to select the appropriate nutritional route and intervention at the appropriate time, based on systematic nutritional assessment and continuous reassessment of the patient's clinical condition.

## The aim of the Research Topic

This Research Topic aims to evaluate the nutritional status of patients across diverse hospital departments, investigate the direct impact of nutritional baseline metrics on clinical outcomes and prognosis, and determine the therapeutic influence of nutritional interventions on the overall patient clinical course.

## Nutritional risk and clinical results of patients in the hospital

This Research Topic represents evidence that nutrition contributes to the clinical evaluation of hospitalized patients and is of importance beyond just identifying malnutrition. In many varied clinical settings, poor nutritional status was found to be connected with increasing rates of mortality, medical complications, decrease in function, and prolonging lengths of stay in the hospital with a greater need for health care. The articles presented in this Research Topic also confirm a continued disconnect between acknowledging nutritional risk in patients and implementing proper nutritional procedures with appropriate and timely medical care.

The difference in risk assessment is clear, especially in general hospitals. A retrospective cohort study by Marinho et al. indicated that within internal medicine units, nutritional risk according to Nutritional Risk Score-2002 (NRS-2002) was detected in 42.4% of patients at the time of admission. However, approximately three out of every four risk patients did not receive any nutritional supplement. Nutritional risk was accompanied by increased mortality, longer hospital stays, and substantially higher hospitalization costs. These findings emphasize that nutritional screening should not represent an isolated administrative procedure, but rather the first step in a structured pathway leading to further assessment and appropriate intervention.

Cao J. et al. reported that patients with comorbid tuberculosis complicated with type 2 diabetes mellitus **(**TB-T2DM) face a high prevalence of nutritional risk, which is driven by both nutritional and immunological indicators. Thus, more attention should be paid to the nutritional status of these patients in order to reduce recurrence and improve prognosis.

Shen et al. utilizing the prospective Investigation on Nutrition Status and Clinical Outcome of Common Cancers (INSCOC) dataset, constructed and validated a novel, cost-effective bedside nomogram that integrates NRS 2002, Triceps Skinfold Thickness (TSF), and pain scores allowing immediate risk stratification for nasopharyngeal carcinoma (NPC) patients.

Regarding the post-operative nutritional status, Chen et al. identified three distinct nutritional status trajectory categories: the “Moderate Malnutrition-Gradual Recovery” group (40.5%), the “Persistent Severe Malnutrition” group (11.7%), and the “Transient Moderate Malnutrition-Rapid Recovery” group (47.9%) in patients after radical surgery for gastrointestinal cancer. In this study, nutritional status was assessed using the Patient-Generated Subjective Global Assessment (PG-SGA).

It should be pointed that secondary to the basic disease malnutrition impacts on the treatment results, infection rate, treatment-related complications, that may lead to prolonged duration of hospitalization. In pediatric patients with disease-related malnutrition (DRM), a single-center prospective cohort study by Garcia-Guzman et al. reported that lower levels of morphofunctional markers, including phase angle (PhA), handgrip strength (HGS), and calf circumference (CC) at hospital admission were related to prolonged hospital stay. The findings of a study by Zhu R. et al. indicated that serum albumin (ALB) levels and the NRS-2002 score could predict prolonged length of hospital stay in patients with upper gastrointestinal (UGI) diseases. These results highlight the importance of nutritional assessment in clinical management and may help guide strategies to improve treatment outcomes in patients. Charle-Cuéllar et al. investigated relapse rates in children treated for severe acute malnutrition (SAM) in Gao, Mali, comparing a simplified protocol to the standard Community Management of Acute Malnutrition (CMAM) approach. Their findings suggest that simplified protocols can reduce relapse rates despite lower ready-to-use therapeutic food (RUTF) doses, though enhanced discharge procedures and post-discharge monitoring remain essential to sustain recovery in emergency settings. Quan et al. indicated that the Peking Union Medical College Hospital individualized fortification (PUMCH IF) improves short-term protein intake and early head circumference growth in preterm infants without increasing feeding intolerance or adverse events.

Regarding critically ill patients' population hospitalized in the Intensive Care Unit (ICU), in a study by Luo et al., the rate of ICU-associated weight change was independently associated with mortality in critically ill patients and demonstrated a non-linear relationship, with a threshold effect at approximately 5%. According to authors, dynamic assessment of weight changes appeared to provide greater prognostic value than static anthropometric measures and may represent a simple and effective bedside tool for mortality risk assessment.

The potential clinical relevance of such intervention is supported by observations in patients with acute-on-chronic liver failure. According to study by Ma et al., more than half of these patients were identified as being at nutritional risk, which was accompanied by longer hospitalization and higher rates of infection, hepatorenal syndrome, hepatic encephalopathy, coagulation abnormalities, and mortality. Additional individualized enteral or parenteral nutritional support was associated with lower rates of several clinically important complications, including infection and spontaneous bacterial peritonitis, although an improvement in all-cause mortality was not observed. Importantly, the absence of a mortality benefit does not exclude clinically relevant effects of nutritional support, particularly with regard to complications and functional outcomes.

Wang Z. et al. analyzed nutritional literacy in older patients with chronic diseases and reported that it was moderately low and heterogeneous, comprising three distinct profiles as follows: “passive dependent type” (23.1%), “cognitive fluctuation type” (42.4%), and “autonomous management type” (34.5%). Interventions should therefore be tailored to individual needs, with particular education and support for the “passive dependent” group to improve self-management, nutritional status, disease control, and quality of life.

## Beyond malnutrition: nutritional, inflammatory, and metabolic risk stratification

A recurring theme across the Research Topic is the increasing use of nutritional and immunoinflammatory indices for prognostic stratification. Routinely available laboratory parameters may capture not only nutritional depletion but also systemic inflammation, immune dysfunction, metabolic reserve, and disease severity. Their clinical relevance was demonstrated across respiratory disease, critical illness, vascular disease, and populations requiring long-term artificial nutrition.

According to Li S. et al., in elderly patients hospitalized with acute exacerbation of chronic obstructive pulmonary disease (COPD), a CONUT score (based on serum albumin, total lymphocyte count, and total cholesterol) ≥5 was associated with a more than 2-fold increase in the adjusted risk of 3-year all-cause mortality. In a multicenter, cross-sectional study, Xu et al. reported that the interaction between nutritional and inflammatory status was also relevant to cardiovascular complications of COPD: lower Advanced Lung Cancer Inflammation Index values and higher CONUT scores were independently associated with atrial fibrillation, while their combination provided additional discriminatory information beyond conventional clinical variables.

Comparable associations were observed in critically ill populations. According to Shao et al., the Geriatric Nutritional Risk Index (GNRI) was independently related to 28-day ICU and in-hospital mortality in patients with sepsis-associated pneumonia and added prognostic information to conventional critical illness severity measures. Similarly, the creatinine-to-albumin ratio, integrating renal dysfunction with a parameter closely related to nutritional and inflammatory status, identified patients with sepsis and diabetes at particularly high short-term mortality risk in a retrospective cohort study by Zhou J. et al. According to Liu R. et al., the modified Geriatric Nutritional Risk Index (mGNRI) is a significant inverse predictor of short-term mortality in urosepsis patients. This practical stratification tool allows clinicians to identify high-risk individuals early and implement targeted interventions.

In a study by Zhai et al., both the CONUT score and PNI were independently associated with an increased risk of sepsis in emergency trauma patients. Thus, these indices may support early risk stratification.

The relationship between nutritional vulnerability and loss of muscle mass further illustrates the multidimensional nature of nutritional assessment. In a retrospective study by Yao et. al., in patients with abdominal aortic aneurysm, NRS-2002, CONUT, and GNRI were independently associated with computed tomography (CT)-assessed sarcopenia. However, differences in sensitivity and specificity limited their usefulness as independent screening instruments for sarcopenia. Thus, nutritional screening and direct assessment of muscle health appear complementary rather than interchangeable.

The prognostic implications of nutritional vulnerability become particularly apparent in older patients with severe dysphagia requiring artificial nutrition. In this population, several complementary indices integrating nutritional, inflammatory, and immune components were associated with long-term outcomes. Higher the C-reactive protein-albumin-lymphocyte (CALLY) values were associated with longer overall survival according to Pan et al., while a higher lymphocyte-to-C-reactive protein ratio (CAR) was similarly associated with lower long-term mortality according to Zhong et al.

A broader comparison of albumin-related indices, performed by Wang and Pan, further demonstrated that higher CALLY, PNI, and lymphocyte–albumin (LA) values were associated with lower overall and infection-related mortality, whereas higher CONUT scores indicated greater risk. Importantly, several of these indices added prognostic information beyond albumin alone. In a study by Liu, Chen et al., body-composition-related measures added another dimension: age- and sex-adjusted adiposity estimators were associated with mortality, particularly pneumonia-related mortality, among older patients receiving percutaneous endoscopic gastrostomy (PEG) and/or total parenteral nutrition (TPN). Together, these studies indicate that nutritional risk in older patients with severe dysphagia is unlikely to be adequately represented by albumin or any other single biochemical marker.

## Nutritional support in critical illness: from initiation to individualized delivery

The articles focusing on critical illness demonstrate that effective nutritional support requires substantially more than the initiation of enteral feeding. Timing, gastrointestinal tolerance, hemodynamic stability, energy adequacy, and avoidance of both insufficient and excessive nutrient delivery must be considered throughout the course of treatment.

Real-world data from respiratory intensive care units highlight the importance of matching nutritional delivery to individual metabolic requirements. A prospective, observational study (ORIENT) by Huang et al. indicated that although achieving nutritional targets remains an important objective, excessive energy delivery may also have adverse consequences: overfeeding was associated with a higher risk of non-social infections and, among patients aged >50 years, with increased 28-day mortality. These findings reinforce a broader principle emerging from the Research Topic: optimal nutritional support should not be equated with maximal nutrient delivery.

Regarding protein supplementation, a meta-analysis and umbrella review of existing evidence by You et al. indicated that compared with conventional protein supplementation, high-protein supplementation does not appear to significantly improve clinical outcomes in critically ill patients, while prolonged high protein intake may be associated with increased overall mortality.

Feeding intolerance continues to be the biggest impediment to adequate enteral feeding in the ICU, and a number of papers have addressed this critical issue from multiple angles. According to Cao X. et al., the use of Acute Physiology and Chronic Health Evaluation II (APACHE II) score, mechanical ventilation, albumin levels, intra-abdominal pressure (IAP), and timing of enteral feeding (EN) in predicting feeding intolerance with EN as the outcome has been shown to have good discrimination. A meta-analysis by Jia et al. revealed that gastrointestinal ultrasound (GI-US) may provide a valuable new objective means of assessing gastrointestinal function using gastric antrum cross sectional area for predicting feeding intolerance. Furthermore, it accelerates the initiation of enteral nutrition without compromising patient safety, establishing it as the superior clinical option.

However, the large number of available prediction models contrasts with limited evidence of their external validity. An overview of systematic reviews by Zhou Z. et al. identified 115 models for enteral feeding intolerance, but fewer than one-third had undergone external validation, while the overall risk of bias was high. Further studies should focus primarily on external validation and clinical usefulness of existing models. According to He C. et al., the lack of core outcome sets had led to significant variability and non-standardization in the outcome indicators reported by Enteral Feeding Intolerance clinical studies. Enteral Feeding Intolerance is an important factor affecting the prognosis of critically ill patients, and the outcome indicators of its clinical studies need to be standardized. It is recommended that a core outcome set for Enteral Feeding Intolerance be constructed to standardize the reporting of outcome indicators in future studies, reduce inter-study heterogeneity, and improve the utility of study results and the quality of clinical decision-making.

Early enteral nutrition was feasible in pediatric patients receiving extracorporeal membrane oxygenation (ECMO) despite the need for hemodynamic support. In a prospective observational study by Ren et al., vasoactive-inotropic therapy itself did not prevent initiation of enteral nutrition within 48 h, although greater vasoactive requirements were associated with poorer achievement of energy targets. These findings indicate that vasoactive-inotropic treatment alone should not preclude early EN, although feeding may need to be advanced more cautiously in patients with higher vasoactive requirements. In a retrospective observational study by Mo et al., early EN (EEN) was not associated with a reduced risk of 28-day mortality in patients with septic shock, although a potential benefit was observed among those receiving low-dose vasopressor therapy. Yuan R. et al. evaluated an evidence-based enteral nutrition management protocol guided by the ACE Star model for neurocritical care patients. The standardized protocol successfully reduces feeding intolerance, enhances nutritional status, and supports patient recovery.

Tambaro et al. retrospectively evaluated the impact of three nutritional source modalities, as oral nutrition (ON), enteral nutrition (EN), and parenteral nutrition (PN) on nutritional status in 125 children undergoing allogeneic hematopoietic stem cell transplantation (allo-HSCT) in two Italian pediatric centers. Nutritional support type significantly influenced BMI changes and hospital stay length, but not clinical outcomes like severe acute graft-vs.-host disease (aGvHD) or engraftment times. Enteral nutrition (EN) minimized BMI loss and led to the shortest hospitalization. Conversely, TPN caused the greatest body mass index (BMI) decline, while ON yielded intermediate results.

Regarding the route of EN, Yuan Y. et al. indicated that the electromagnetic navigation-guided placement (ENG-P) technique outperforms conventional blind insertion method for NJT placement (CBN-P) for bedside nasojejunal tube (NJT) placement in the ICU by offering higher first-attempt success, faster procedures, fewer reinsertions, and reduced costs.

Regarding the optimal EN delivery strategy, a meta-analysis by Hu et al. indicates that continuous enteral nutrition (CEN) appears superior to intermittent enteral nutrition (IEN) in critically ill patients, particularly those requiring mechanical ventilation. In this study, IEN was associated with an increased incidence of diarrhea and abdominal distension, higher ICU mortality, and prolonged length of ICU stay. These findings support the preferential use of CEN in ventilated patients while emphasizing the need for individualized nutritional management based on gastrointestinal tolerance.

## Parenteral nutrition: indications, modalities, safety, and clinical applications

Parenteral nutrition (PN) is required when nutritional requirements cannot be met through the oral or enteral route. Evidence synthesized for hospitalized oncology patients by Liu, Liu et al. identified 46 evidence items spanning individualized nutritional decision-making, prescription and review, preparation and compounding, administration and monitoring, and safety and quality management. The findings emphasize the role of multidisciplinary Nutrition Support Teams and institutional quality procedures in the safe use of PN. Regarding the route of long-term PN, in a retrospective study by Rong et al., including 73 patients aged ≥65 years with dysphagia receiving long-term PN via implantable central venous access port (ICVAP), non-tunneled central venous catheter (NT-CVC), or peripherally inserted central catheter (PICC), patients using ICVAP demonstrated improved survival outcomes compared to those with NT-CVC, whereas no meaningful survival disparity was found between the PICC and ICVAP cohorts.

## Metabolic, perioperative, and micronutrient dimensions of hospital nutrition

Several contributions expanded the concept of nutritional care beyond conventional malnutrition and artificial feeding by examining metabolic responses, micronutrient status, and fluid balance.

Perioperative nutritional management may influence metabolic recovery even in patients with established metabolic disease. In a randomized control trial by Wang C. et al., preoperative oral isomaltulose in patients with type 2 diabetes undergoing elective surgery reduced post-operative insulin resistance and glycemic variability without delaying gastric emptying, although transient increases in pre-induction glucose highlight the need for individualized perioperative carbohydrate strategies.

Metabolic indices derived from routine clinical variables may also provide early prognostic information. A higher triglyceride-glucose-body mass index (TyG-BMI) was associated with increased risk of early-onset acute kidney injury (AKI) among critically ill patients, and its incorporation into conventional models improved risk reclassification in a retrospective study by Zhu Q. et al. Dynamic fluid status represents another important component of critical-care management: Zhang Q. et al. reported that after cardiac arrest, a trajectory characterized by relatively rapid transition toward a moderately negative fluid balance was associated with the most favorable prognosis, whereas more persistent positive fluid balance trajectories were associated with increased mortality.

Micronutrient status may similarly identify clinically vulnerable patients. Vitamin D (VD) is a fat-soluble vitamin and secosteroid prohormone that plays an important role in calcium and phosphate homeostasis as well as in a wide range of physiological processes (58, 59). In a study by Hung et al., vitamin D deficiency preceding ischemic stroke was associated with a higher risk of acute kidney injury as well as mortality, ICU admission, pneumonia, and dialysis requirements. A study by Shi et al. indicated that in chronic spinal cord injury, vitamin D deficiency or insufficiency was highly prevalent and lower 25(OH)D concentrations were strongly associated with neuropathic pain. However, both studies were observational. Therefore, these findings should not be interpreted as evidence that vitamin D supplementation would improve the reported clinical outcomes. Feng et al. reported that vitamins A and D deficiencies in geriatric sepsis patients inversely correlated with procalcitonin (PCT) and interleukin-6 (IL-6) levels. Additionally, a deficiency in either vitamin serves as an independent risk factor for a higher prevalence of sepsis in this population.

## Broadening nutritional care toward patient-centered and precision approaches

The Research Topic also highlights dimensions of nutritional care that are easily overlooked by conventional screening instruments. Nutritional intake and recovery depend not only on physiological requirements but also on sensory function, psychological state, motivation, and patient engagement. Among liver transplant recipients, observed by Cinar et al., malnutrition frequently coexisted with severe disturbances in smell and taste, while greater sensory impairment and lower hedonic pleasure were associated with more severe malnutrition. In elderly hospitalized patients, analyzed by Li L. et al., nutrition awareness was related to death anxiety and future time perspective, suggesting that psychological adaptation and perception of the future may influence engagement with nutritional health behaviors. Together, these findings encourage a broader understanding of nutritional care that incorporates the patient's experience alongside biological measures.

At the same time, several contributions point toward a transition from conventional nutritional screening to multidimensional and increasingly individualized risk prediction. He Z. et al. reported that in older adults undergoing surgery for hip fracture, machine-learning approaches integrating demographic, clinical, nutritional, and psychological variables demonstrated good performance in identifying Global Leadership Initiative on Malnutrition (GLIM)-defined perioperative malnutrition. According to Zhang C. et al., among malnourished elderly ICU patients, nutritional vulnerability could similarly be incorporated into prediction of delirium: GNRI was retained alongside markers of disease severity and physiological instability in an externally validated machine-learning model.

Combining inflammatory and nutritional information may improve clinical risk assessment. A retrospective multicenter cohort study by Cai et al. indicated that in patients with traumatic brain injury (TBI), a machine-learning model combining clinical variables with the Pan-Immune-Inflammation Value (PIV) and PNI successfully stratified the risk of hospital-acquired pneumonia (HAP), with PIV and PNI emerging among the most influential predictors. These results suggest that combining nutritional and inflammatory markers with conventional clinical variables may improve risk stratification, although the clinical value of such integrated models requires further validation.

Overall, the studies included in this Research Topic demonstrate that nutritional risk in hospitalized patients is closely linked to clinical outcomes across a wide range of conditions. They also show that identifying nutritional risk is not sufficient if screening is not followed by appropriate assessment and intervention. At the same time, nutritional status cannot be considered in isolation from inflammation, muscle mass, metabolic disturbances, organ dysfunction, and the patient's ability to tolerate nutritional support. Future studies should determine whether the increasingly available prognostic indices, bedside assessment methods, and machine-learning models translate into better nutritional decisions and, ultimately, improved patient outcomes.

## Summary and conclusion

This Research Topic demonstrates that baseline nutritional metrics profoundly impact clinical outcomes, confirming a direct link between poor nutritional status and increased mortality, medical complications, and prolonged hospital stays. The findings emphasize that nutritional screening must evolve from an isolated administrative task into the mandatory first step of a structured clinical pathway integrating timely interventions and advanced risk stratification.
